# A glance at the emerging diagnostic biomarkers in the most prevalent genitourinary cancers

**DOI:** 10.1016/j.sjbs.2022.01.017

**Published:** 2022-01-15

**Authors:** Mohammed Merae Alshahrani

**Affiliations:** Department of Clinical Laboratory Sciences, Faculty of Applied Medical Sciences, Najran University, 1988, Najran 61441, Saudi Arabia

**Keywords:** MRI, Magnetic resonance imaging, PET, Positron emission tomographic, PSA, Prostate-specific antigen, CTLA4, Cytotoxic T-Lymphocyte Antigen 4, sncRNAs, Small non-coding RNAs, SNPs, Single nucleotide polymorphisms, APC, Antigen-presenting cells, TCR, T- cell receptor, MHC, Major histocompatibility complex, IL-2, Interleukin-2, CRP, C-reactive protein, mCRPC, Metastatic Castration-Resistant Prostate Cancer, PSMA, Prostate-specific Membrane Antigen, PCFT, Proton-coupled folate transporter, RFC, Reduced folate carrier, FR, Folate receptor, PI3K, Phosphatidylinositol 3-kinase, CRPC, Castrate-resistant prostate cancer, VHL, Von Hippel-Lindau, VEGF, Vascular endothelial growth factor, CAIX, Carbonic anhydrase IX, CTCs, Circulating tumor cells, Genitourinary cancers, Immunological biomarkers, Tissue molecular biomarkers, Liquid molecular biomarkers, Specific, Sensitive, Less cost

## Abstract

Genitourinary cancers comprise of a heterogenous group of cancers of which renal cell carcinoma, urothelial bladder carcinoma, and prostate adenocarcinoma are the most commonly encountered subtypes. A lot of research is ongoing using various strategies for exploration of novel biomarkers for genitourinary cancers. These biomarkers would not reduce the need for invasive diagnostic techniques but also could be used for early and accurate diagnosis to improve the clinical management required for the disease. Moreover, selecting the appropriate treatment regimen for the responsive patients based on these biomarkers would reduce the treatment toxicity as well as cost. Biomarkers identified using various advanced techniques like next generation sequencing and proteomics, which have been classified as immunological biomarkers, tissue-specific biomarkers and liquid biomarkers. Immunological biomarkers include markers of immunological pathways such as CTLA4, PD-1/PDl-1, tissue biomarkers include tissue specific molecules such as PSA antigen and liquid biomarkers include biomarkers detectable in urine, circulating cells etc.

The purpose of this review is to provide a brief introduction to the most prevalent genitourinary malignancies, including bladder, kidney, and prostate cancers along with a major focus on the novel diagnostic biomarkers and the importance of targeting them prior to genitourinary cancers treatment. Understanding these biomarkers and their potential in diagnosis of genitourinary cancer would not help in early and accurate diagnosis as mentioned above but may also lead towards a personalized approach for better diagnosis, prognosis and specified treatment approach for an individual.

## 1 Introduction

**Fig. 1:**
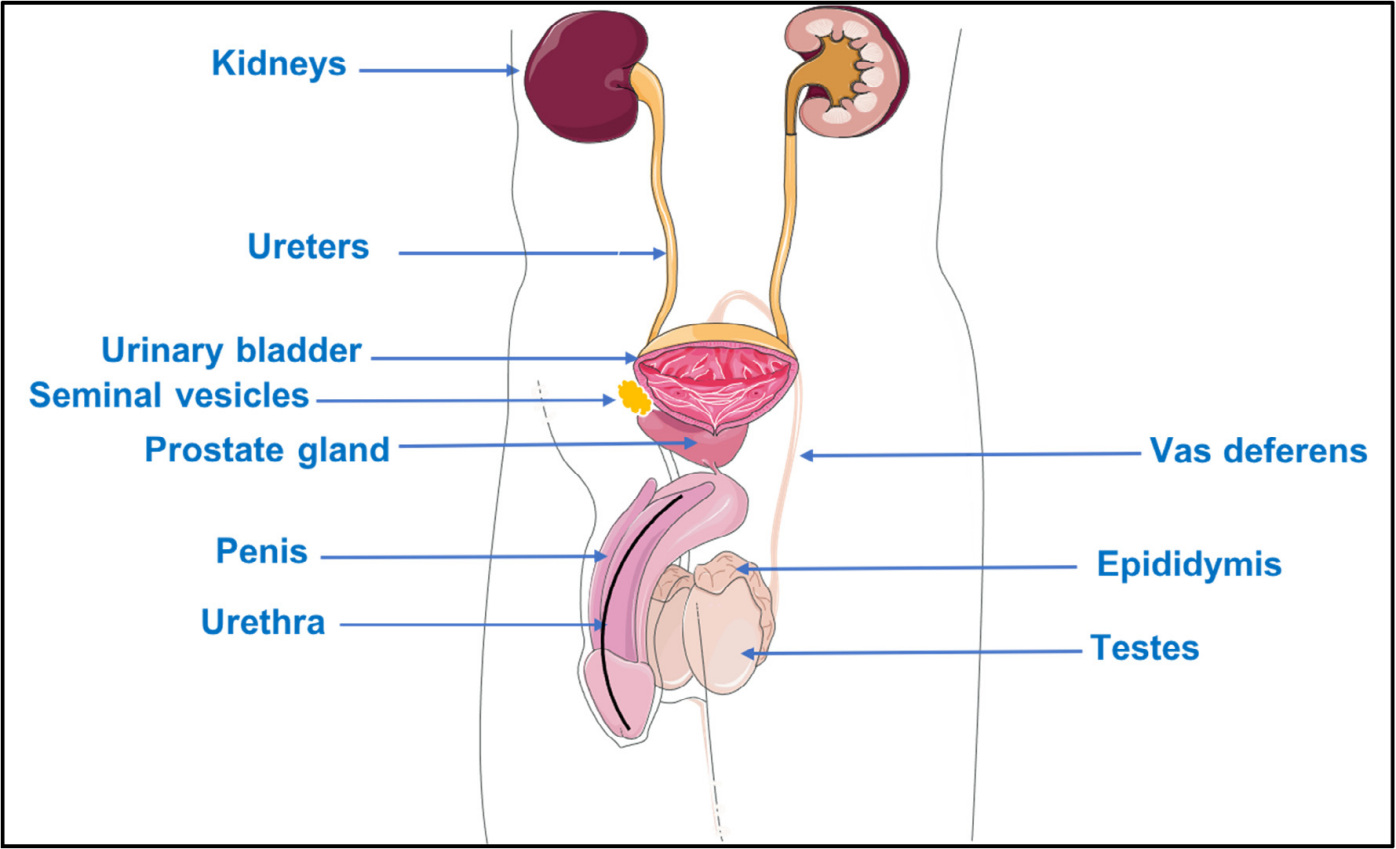
Schematic diagram of the Male Genitourinary System anatomy. Figure generated by (https://smart.servier.com).

The genitourinary system (GUS) cancers are a heterogenous group of malignancies related to specific anatomical and physiological function (Zarrabi et al., 2019). Bladder carcinoma (BC), renal cell carcinoma (RCC), and prostate cancer (PC) are the most common histological subtypes of the GUS cancers (Zarrabi et al., 2019). GUS cancers originate in genitourinary system which consists of two components, the urinary component related to the excretory system and the genital part related to the reproductive system (Groat, 2009, Bankir et al., 1989, Clement and Giuliano, 2015, Bronson, 2011) (Fig. 1).

The total share of GU cancers among all other cancers in both genders is approximately 20.80% (Tolou_Ghamari et al., 2019). A previous study has shown that the urothelial cancers account for more than 90% of the cancers occurring in the urinary tract (Yaxley, 2016). Some of the cancers originating in this system are rare, including squamous cell carcinomas, small cell carcinomas, and some adenocarcinomas (Yaxley, 2016). Cancers rarely occur in ureter and kidney's pelvis, and both account for merely 4% of the urothelial cancers (Tolou_Ghamari et al., 2019). The knowledge regarding the female reproductive system malignancies is more comprehensive. Studies have demonstrated the frequency of cervical cancer to be 66.30%, ovarian carcinoma 21.10%, uterine cancer 9%, and that of the vulva to be 2.60% (Tolou_Ghamari et al., 2019).

## 2 Diagnosis of genitourinary system Cancers:

Most of the available diagnostic and prognostic procedures for GUS cancers are invasive and have a very low sensitivity and specificity. Given the significant illness and mortality rates associated with GU cancers, there is an imperative need for development of clinical non-invasive testing based on biomarkers for early detection of disease along with disease monitoring and treatment response monitoring (Boguslawska et al., 2019). A significant number of GUS cancer patients are typically identified at an advanced stage due to lack of sensitive prognostic biomarkers, and thereby, the 5-year survival rate remains far from ideal (Urquidi et al., 2012). Keeping this in view, researchers around the globe have recently focused on identification of novel tumor biomarkers associated with GUS cancers screening, diagnosis, prognosis, and therapy efficacy evaluation to enhance the survival rate of the patients.

With the advent of novel biomarkers and clinical validation of new diagnostic tools, a rapid change in diagnostic modalities has been seen (Manzi et al., 2020).

In this review, we aim to provide a cumulative update regarding the emerging novel biomarkers for GUS cancer and their potential in diagnosis, follow-up, and treatment response monitoring along with a brief description about types of GUS cancers.

## 3 Classifications of genitourinary cancers

### 3.1 Bladder cancer

Bladder cancer (BC) accounts for sixth most common malignancy in males globally (GLOBOCAN, 2020). Urothelial cancer, one of the most common types of bladder cancer is caused by the invasion of neoplastic cells of urothelial origin into the basement membrane, lamina propria. Bladder cancer is divided into low stage (1 and 2) and a high stage (3), the difference between these stages impacts the risk assessment and patient treatment (Kaseb and Aeddula, 2021). There are several identified risk factors for BC, such as smoking, infection of schistosomiasis, and contact with certain chemicals (Hinotsu et al., 2009, Humphrey et al., 2016, Pelucchi et al., 2006 Ames et al., 1975, Cumberbatch et al., 2016, Gaertner et al., 2004).

### 3.2 Kidney cancer

Kidney cancer is considered the seventh most frequent cancer in Europe, North America, Australia/New Zealand, and Japan (Padala et al., 2020). Kidney cancer incidence rates rise gradually with age, reaching a peak around 75 years (Znaor et al., 2015). Tobacco usage, body mass index, and a history of hypertension and chronic renal disease are known to be the most risk factors causing kidney cancer (Scelo and Larose, 2018; Cumberbatch et al., 2016; Bhaskaran et al., 2014, Wang and Xu, 2014; Hofmann et al., 2015, Shebl et al., 2012; Hofmann et al., 2015, Lipworth et al., 2012). Additionally, in the United States, a history of high blood pressure has been anticipated to increase the chance of kidney cancer (Colt et al., 2011; Sun et al., 2015, Weikert et al., 2008 Chow et al., 2000). Additionally, several case-control studies have found a link between having a history of kidney stones and having a higher chance of developing kidney cancer, while prospective cohort studies have been equivocal (Cheungpasitporn et al., 2015, Shih et al., 2014; Leslie et al., 2021).

### 3.3 Prostate cancer

Prostate Cancer (PC) is considered as the fourth major cause of death globally (Jemal et al., 2010, Mattiuzzi and Lippi, 2019 Leslie et al., 2021; Elmehrath et al., 2021). PCs, for the most part, grow slowly with a low recurrence chance and a low level of aggressiveness (Jemal et al., 2010, Mattiuzzi and Lippi, 2019, Torre et al., 2015). PC is often caused in advanced stage, with incidences as high as 90% in males aged 70 to 90 (Haas and Sakr, 1997; Dunn and Kazer, 2011). Occurrence of PC has been found to be associated with the racial background and ethnic origin (Taitt, 2018; Dunn and Kazer, 2011). Genetics, family history and diet such as increased consumption of red meat, low vitamin D blood level, increased carbohydrate intake, are associated with increased risk of PC (Benafif and Eeles, 2016;Kiciński et al., 2011; Barfeld et al., 2014; Freedland et al., 2008, Lin et al., 2015, Schenk et al., 2014, Wilson et al., 2016). Role of various genes in occurrence of PC has been deciphered such as mutations in BRCA1 and BRCA2 (Tan et al., 2018).

## 4 Methods of diagnosis of genitourinary cancers

Diagnostic methods used for GU cancers depend on the type and location of the suspected GU cancer. Other factors affecting the selection of the diagnostic procedure include patient's pertinent history, findings from the clinical examination, and the patient's overall health state (Roobol et al., 2010). Imaging systems such as computed tomographic (CT), magnetic resonance imaging (MRI), ultrasonography, positron emission tomographic (PET) scanning, intravenous pyelography, and angiography are the conventional diagnostic modalities for the GU malignancies (O’ Donoghue et al., 2010).

Other diagnostic techniques include endoscopic examination using an endoscope to visualize the urinary tract (Huffman and Clayman, 1985). Furthermore, a tissue biopsy can also be used to detect cancer for establishing a pathological diagnosis (Getzenberg et al., 1996). Additionally, molecular techniques such as analysis of the DNA for detection of various tumor-specific genes and other molecular-level findings are also in use (Netto and Cheng, 2012).

Conventional cystoscopy uses white light for diagnosing BC. However, it fails to distinguish between benign and malignant growths (Herr, 1999, van der Poel and Debruyne, 2001). To overcome this limitation, blue-light cystoscopy is used to illuminate cancerous growths and differentiate malignant tumors from the benign ones (Hermann et al., 2011). The diagnosis of PC is quite different from that of all other GU cancers. It uses many additional diagnostic methods compared to other GU cancers. For instance, digital examination of the rectum, ultrasound-guided biopsies, MRI-guided biopsies, and diagnostic biomarkers such as the prostate-specific antigen (PSA) (Descotes, 2019).

Identification of biomarkers in GUS tumors has gained a lot of attention as this is the most feasible diagnostic and prognostic strategy as discussed above. This search for novel biomarkers progressed due to the development of new and emerging analysis approaches for identification of biomarkers. With the use of mass-spectrometry, various biomarkers that can better diagnose various GU cancers, including kidney, bladder, and prostate cancers have been explored (Chen et al., 2019). Proteomics approach coupled with multiplexed quantification assays is one of the techniques implicated for identification of biomarkers using serum, urine and tissue samples (Zhang et al., 2017). In a recent study, aberrant plasma profile of patients with low grade non-muscle invasive BC and healthy patients was studied to identify biomarkers for BC. Among many other identified proteins, haptoglobin was found to be differentially expressed based on 2D-DIGE and mass-spectrometry analysis. Further analysis revealed that haptoglobin was able to distinguish between low grade BC and controls with high sensitivity and specificity (Nedjadi et al., 2020). However, more extensive validation studies are needed for clinical implication of findings of such studies. Manzi et al., in their review, have provided a detailed information regarding capabilities of the mass spectrometry based oncometabolomics approach for improving the detection, early diagnosis, risk stratification and evaluation of the outcome (Manzi et al., 2020). Immune system has a pivotal role in modulating the immune response to various targeted therapeutic agents. Based on this, various promising immunotargets such as such Cytotoxic T-Lymphocyte Antigen 4 (CTLA4), programmed death 1 (PD-1) and PD ligand 1 (PD-L1) and therapeutic strategies based on these targets have been evolved. Various studies have been conducted to evaluate the prognostic, predictive and therapeutic role of these molecules in patients with RCC, BC, and PC (Lopez-Beltran et al., 2018). Data of Checkmate 025 trial assessing the efficacy of nivolumab in patients showed that expression of PDL-1 had a more prognostic role than a predictive role. Role of these immunological biomarkers have been detailed in upcoming section. Role of various genes related to immune signaling pathways such as KDM5C, PTEN, MTOR and TP53 as biomarkers for GUS specially RCC has been studied but no conclusive data is still available (Motzer et al., 2020; Zhao et al., 2019). DNA and RNA based genome analysis have also been used for identification of genetically variant biomarkers for GU cancers. The advent of next generation sequencing has made the identification of genetical variants in solid tumors very feasible. In their review, Giunchi et al., have provided comprehensive information regarding these molecular targets/biomarkers for GU cancers (Giunchi et al., 2018). They have mentioned that analysis of BRCA1 and BRCA2 germ-line mutations could be much use for not only risk assessment in case of PC but also to predict the response to the treatment with PARP-inhibitors. Sequencing of cancers have also revealed that assessing the tumor mutation burden based on the number of mutations in tumor cell DNA has also become one of the most relevant predictor of responses to immunotherapy targeting PD-1/PDL-1 axis (Han et al., 2020). Not only DNA based genome sequencing, but RNA based genome sequencing has been widely used in GU cancers for identification of biomarkers which could prognostic and therapeutic implications for patients with GU cancers (Robertson et al., 2017). Expression of Small non-coding RNAs (sncRNAs) was analyzed by Sabo AA et al. in extracellular vesicles in BC by next-generation sequencing and it was found that sncRNAs present in plasma extracellular vesicles such as miR-4508, miR-126-3p, miR-185-5p, miR-106a-5p, and miR-10b-5p, could serve as diagnostic markers for BC (Sabo et al., 2020).

Most of the above-mentioned studies have focused on one or the other type of biomarkers explored in case of GU cancers. Herein, we are attempting to provide the comprehensive information about various biomarkers and strategies used to explore them on one platform which has been discussed in sections below.

## 5 Emerging diagnostic biomarkers

### 5.1 Introduction to biomarkers

The presence of biomarkers in a tissue or a body fluid depicts the presence of a specific disease or disorders including cancer. Biomarkers play an important role in oncology. A particular biomarker present in a specific disease imparts a diagnostic caliber to the biomarker for that disease. This makes biomarkers suitable for their diagnostic or prognostic role in various types of malignancies (Micheel et al., 2012).

The discovery of biomarkers for GU cancers is the subject of interest for researchers all over the world. The growing interest in GU tumors is primarily due to the clinicians' desire to limit the use of various invasive methods of diagnosis due to the adverse effects. The other reasons include improving the patient outcomes who are likely to respond well to therapy, curtailing the toxic effects of chemotherapy, and cut the economic cost resulting from treatment to which the patients are refractory (Chen et al., 2019, Montironi et al., 2019).

In last couple of years, since the discovery of biomarkers has been implicated in the field of oncology, they have become a priority area of cancer research. For instance, the use of PSA as a biomarker to screen prostate cancer in masses has provided much motivation to the investigators to research biomarkers that can play similar role in managing other cancers (Alford et al., 2017).

### 5.2 Uses and roles of biomarkers in oncology

The general uses of biomarkers in oncology include assessing cancer risk, screening, establishing differential diagnoses, determining the disease prognosis, predicting the response to therapy, and monitoring cancer progress (Ludwig and Weinstein, 2005). It is pertinent to note that biomarkers have their role in all the stages of cancer. However, to use their role in all the stages of the disease, there is a need of comprehensive evaluation for the clinical validity and utility of the identified biomarkers in tracking the progression of the disorders (Henry and Hayes, 2012). These multiple applications of cancer biomarkers in oncology could have a significant and positive impact in cancer treatment (Henry and Hayes, 2012).

The role of biomarkers in cancers is not limited to disease diagnosis and prognosis only. Biomarkers find their application in various other clinical scenarios as well. For example, they are used for the estimation of the risk of diseases, searching for occult cancerous growths, discriminating between both the benign and malignant lesions, differentiating various types of cancers of the same tissue/organ, and for the detection of cancer recurrence (Santosh et al., 2016). Biomarkers, therefore, have varied roles in managing various types of cancers, including GU cancers (Franzen et al., 2016).

## 6 Emerging biomarkers in the genitourinary cancers

**Fig. 2:**
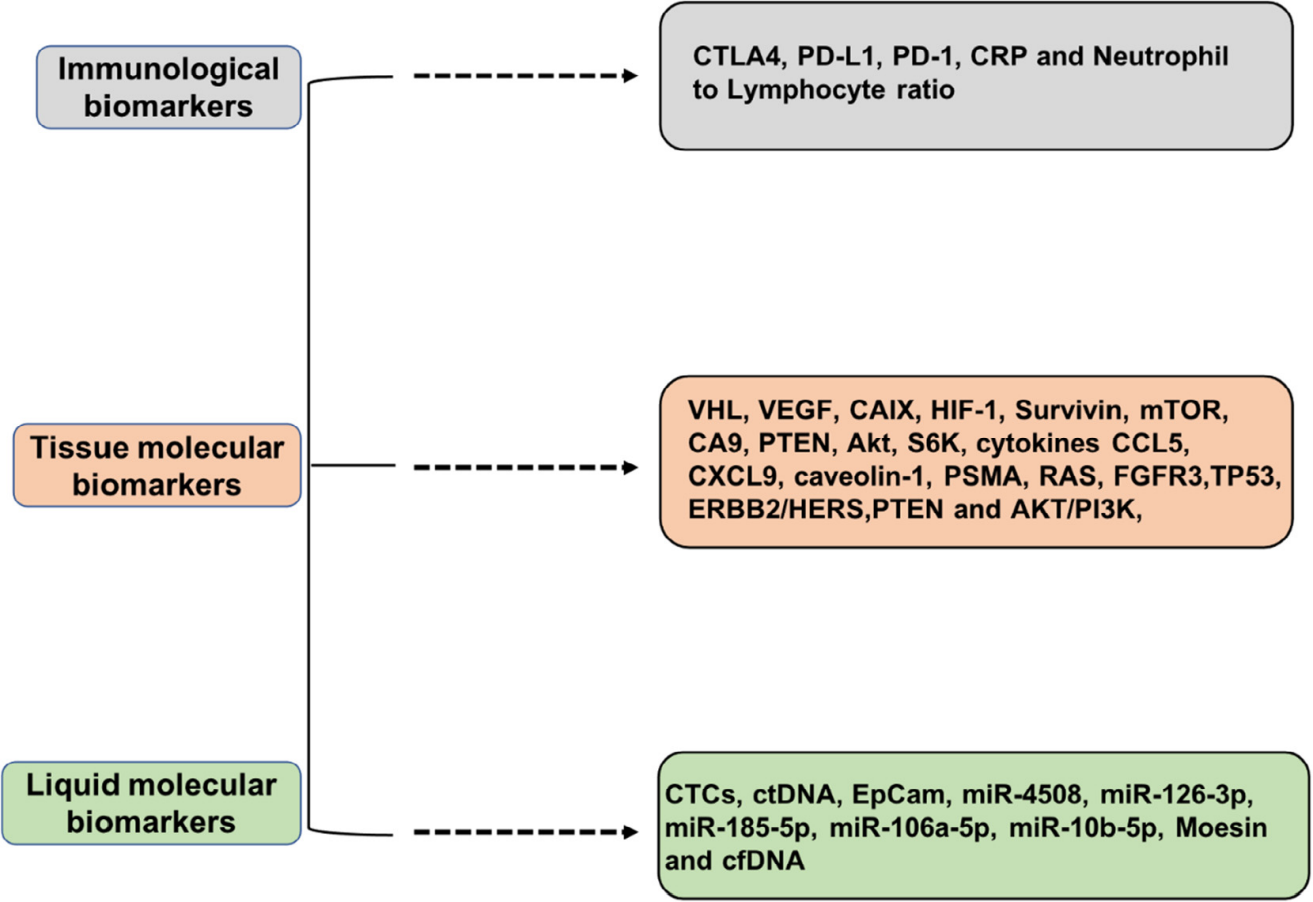
A schematic diagram showing the types of biomarkers that can assist in the detection of genitourinary cancers at early stages and help improve targeted therapies based on them.

Biomarkers used for the diagnosis of GU cancers have been highly evolved. Many biomarkers are emerging to promise a transformation in the diagnosis and treatment of these cancers in the current times. The emerging biomarkers in GU cancers can be classified as immunological, tissue molecular, and liquid molecular biomarkers (Fig. 2) (Montironi et al., 2019).

### 6.1 Immunological biomarkers in genitourinary cancers

**Fig. 3:**
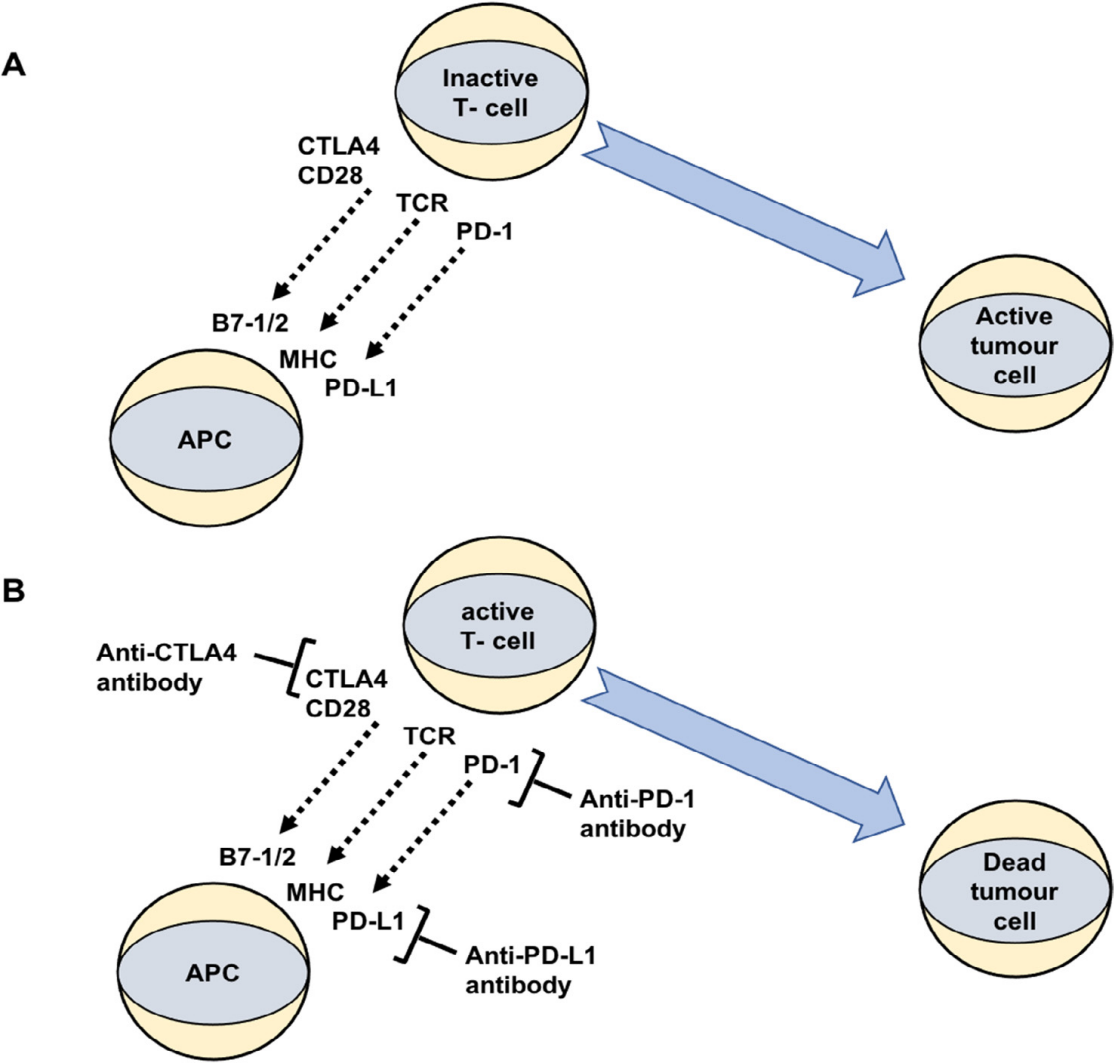
Mechanism of tumor-specific T cells generation. B7-1/2 and PD-L1 on antigen-presenting cells (APC) and CTLA-4 on T cells are checkpoint proteins that help keep the body's immunological responses in control. T cells are activated when the T- cell receptor (TCR) attaches to antigen and major histocompatibility complex (MHC) proteins, CD28 binds to B7-1/2 and PD-L1 to PD-1 on the APC. In tumor cases, the binding of B7-1/2 to CTLA-4 maintains T cells in an inactive state, preventing them from destroying tumor cells (A). Targeting CTLA-4, PD-1and PD-L1 with immune checkpoint inhibitors (anti-CTLA-4, anti-PD-1, and anti-PD-L1 antibodies) resulted in T cells becoming activated and can destroy tumor cells (B). Figure adapted and modified from (Andrews et al., 2019).

Immune cells perform a significant role in the pathogenesis, cellular multiplication, and metastasis of GU tumors. Immune system of humans has the capability to modulate the response to chemotherapeutic drugs (Markman and Shiao, 2015). It Numerous immunotherapeutic strategies have been developed to explore diverse immunotargets with the potential of cancer treatment (Montironi et al., 2016). Some of these immune targets include CTLA4, PD-1, and the PD-L1 (Kassardjian et al., 2018). CTLA4 is an immunoregulatory protein that inhibits antitumor responses by inhibiting T cell activation (Fig. 3A) (Andrews et al., 2019). Candidate disease-susceptibility single nucleotide polymorphisms (SNPs) of the CTLA4 gene have been investigated in BC patients from the North Indian community. The CTLA4 mutation genotype revealed a 3.74-fold increase in the incidences of BC, suggesting that genetic differences in the CTLA4 gene are associated with BC susceptibility (Jaiswal et al., 2014).The upregulation of CTLA4 pathway was also found to be significant among the young cohort of prostate cancer patients and was linked to the biochemical recurrence (Ding et al., 2016). Furthermore, overexpression of the CTLA4 gene was also detected in RCC patients, which was correlated with poor overall survival rate (OSR), progression-free survival (PFS), and disease-free survival (DFS) in these patients (Liao et al., 2021).

One of the other important and well explored immune targets is PD-L1 which binds to PD-1 on lymphocytes and initiates signalling pathway for prevention of T-cell receptor (TCR)-mediated activation and production of interleukin-2 (IL-2) and T-cells. The interface between PD-L1 and PD-1 is critical for establishing peripheral immunological tolerance, which is used by cancer cells to evade antitumor immunity (Fig. 3B) (Andrews et al., 2019, Dong et al., 2002).

According to a recent analysis in Indian population, PD-L1 was found to be upregulated in RCC patients and was accompanied by an elevated risk of tumor-specific recurrence and mortality. It was also linked to a higher stage at the first presentation, indicating more aggressive tumors (Kumar et al., 2019). Interestingly, PC has a greater prevalence of PD-L1 expression than PD-1, which may be considered as a co-factor in PC progression (He et al., 2021).

On the other hand, it has been found that BC patients showed overexpression of both PD-1 and PD-L1, which was associated with an improved survival rate due to higher immune competence (Breyer et al., 2018). Contrary to the above finding, immunohistochemistry study of formalin-fixed paraffin-embedded urothelial tumor samples showed that PD-L1 expression level in cancer cells is not linked with the survival rate except those with metastatic BC (Bellmunt et al., 2015). Recently, in high-grade T1 bladder tumors, PD-L1 was shown to be extensively expressed on immune cell infiltrates but not on the tumor cells, and there was no connection between PD-L1 and outcomes (Sam et al., 2017). It cannot be over-ruled that some of the differences among the findings of ese studies may be due to the use of various PD-L1 antibodies, quantification techniques, tumor features, and cell types (Yu and Rao, 2019). Still due to so many contrary findings, the prognostic role of PD-L1 expression in BC remains unclear (Yu and Rao, 2019).

There are many immunological biomarkers available in routine clinical practice, such as C-reactive protein (CRP) and neutrophil-to-lymphocyte ratio (Gaudreau et al., 2016). CRP is a pentraxin family member that plays a role in carcinogenesis and inflammation as well as has a predictive value in diverse stages of PC (Gaudreau et al., 2016). Elevated CRP was linked to OSR and clinical DFS in patients with localized PC treated with radiation (Thurner et al., 2015). On the other hand, during therapy for metastatic castration-resistant prostate cancer (mCRPC), a high ratio of neutrophil-to-lymphocyte has a predictive value (De Giorgi et al., 2015).

### 6.2 Tissue molecular biomarkers

The ever-increasing cases of GU cancers, especially PC, have intensified the need for improving diagnostic, imaging, and treatment strategies. The Prostate-specific Membrane Antigen (PSMA), which is a tissue-based molecular biomarker, is intensely expressed in advanced cases of PC (Ross et al., 2003). This antigen has demonstrated the possibility of serving as an effective target for the diagnosis and therapy for PC (Bouchelouche et al., 2010).

**Fig. 4:**
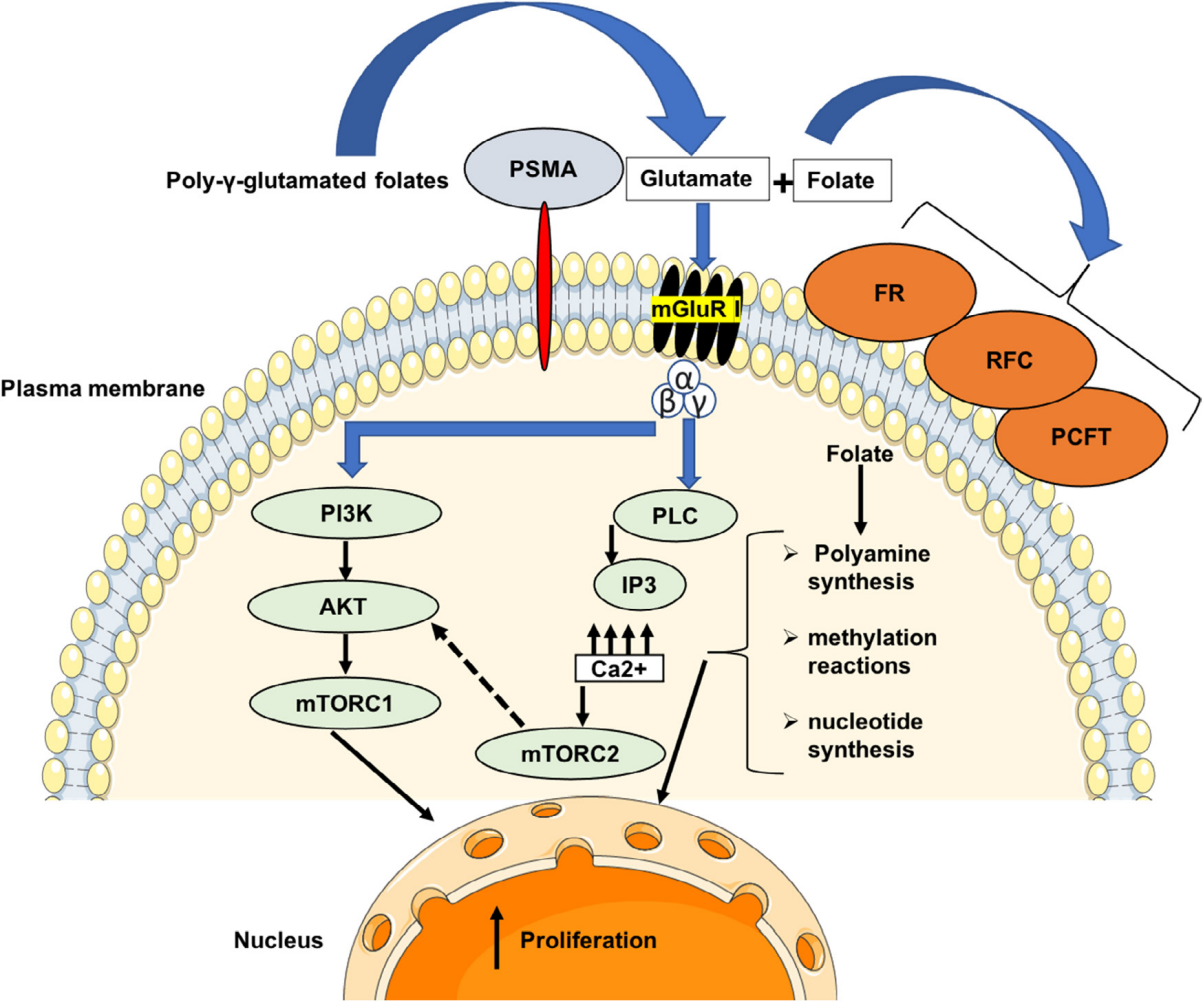
The role of PSMA in tumorigenesis. PSMA hydrolyses poly—glutamated folates produced by dead and dying tumor cells to folate, which is subsequently taken up by surrounding healthy tumor cells through the proton-coupled folate transporter (PCFT), folate receptor (FR), or reduced folate carrier (RFC). Once within the cell, folate is polyglutamated again and utilized for polyamine synthesis, methylation processes, and nucleotide synthesis, all essential for cell motility, including migration, invasion, and proliferation. In addition, PSMA hydrolysis also yields glutamate, which activates the mGluR I receptors on the prostate cancer cells' plasma membranes. Glutamatergic system activation causes calcium signaling and the PI3K cascade to be activated, which controls tumor development. Figure adapted and modified from (Kaittanis et al., 2018, O’Keefe et al., 2018).

PSMA converts polyglutamated folates produced by dying tumor cells to folate, which is then absorbed by surrounding healthy cancer cells through the proton-coupled folate transporter (PCFT), reduced folate carrier (RFC), or folate receptor (FR). When folate enters the cell, it is polyglutamated and utilized for the production of polyamine and nucleotide and methylation processes, all of which are vital for cellular motility, including migration, invasion, and proliferation (Fig. 4) (O’Keefe et al., 2018). Additionally, PSMA hydrolysis results in the production of glutamate, which stimulates the activation of the mGluR I receptors localized on the plasma membrane of the prostate tumor cells. The stimulation of the glutamatergic pathway results in the motivation of calcium signaling and the activation of the phosphatidylinositol 3-kinase (PI3K) pathway, which in turn regulates tumor formation (Fig. 4)(Kaittanis et al., 2018).

An experimental study on prostate and adjoining lymph node samples from the prostatectomy demonstrated that the expression of PSMA on immunohistochemistry enhanced steadily from a focal pattern in the intact prostate gland to a diffuse pattern in the secondary lesions (Ferraro et al., 2020). Another analysis revealed an abrupt rise in the expression of PSMA on immunohistochemistry in contrast with prior benign PC, and its expression was observed in metastatic bone lesions in castrate-resistant prostate cancer (CRPC) (Schmittgen et al., 2003). All these studies established that PSMA expression changes in PC and can therefore, serve as a potential diagnostic marker for PC.

**Fig. 5:**
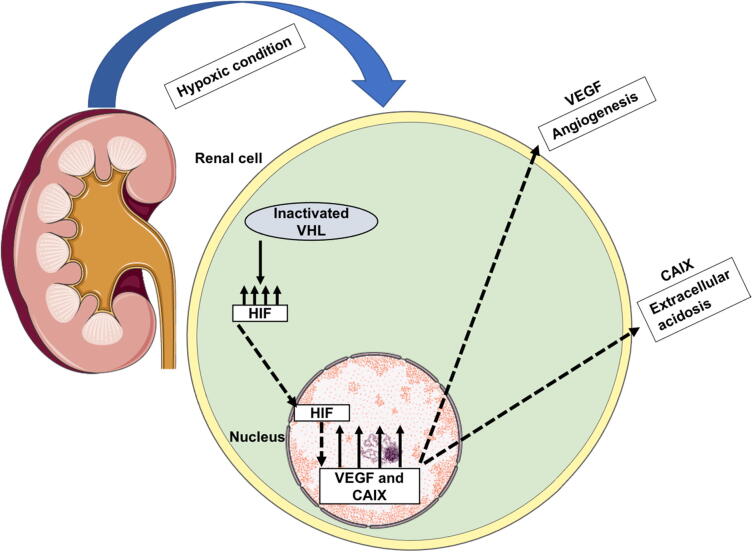
The role of VHL, VEGF, and CAIX biomarkers in tumor angiogenesis. In order to degrade hypoxia-inducible factor (HIF), the VHL gene carries the von Hippel-Lindau protein must be expressed. The HIF protein is often overexpressed in tumors when hypoxia is present. Under hypoxic circumstances, when VHL is inactivated, the HIF protein cannot be destroyed, and hence its level rises even more. Tumor cells with activated HIF undergo nuclear translocation, resulting in transcription of a wide range of genes such as the VEGF that play a potent role in tumor angiogenesis. Additionally, in order to protect cancer cells from intracellular acidosis and permit rapid tumor development, overexpression of CAIX causes extracellular acidosis, which stimulates cancer cell motility, including migration, invasion, and proliferation. Figure adapted and modified from (Choueiri and Kaelin, 2020, Lee and Griffiths, 2020).

For RCC, the three major predictive and prognostic indicators are Von Hippel-Lindau (VHL) tumor suppressor gene, vascular endothelial growth factor (VEGF), and carbonic anhydrase IX (CAIX) enzyme (Fig. 5) (Garcia et al., 2009, Sültmann et al., 2005). It has been revealed that the I–III stage of clear cell RCC patients managed by nephrectomy showing VHL changes had better outcomes (Yao et al., 2002). Furthermore, patients with VHL gene alterations responded better to anti-VEGF treatment than those with the wild-type gene (Choueiri et al., 2008).

VEGF is a key ligand in the tumor angiogenesis (Fig. 5) (Choueiri and Kaelin, 2020). Treated patients with anti-VEGF therapy showed a decreased level of soluble VEGFR and increased level of serum VEGF (Garcia et al., 2009). In another study, these factors' concentrations in the blood were monitored both before and after RCC patients received VEGF-targeted treatment. RCC patients with high serum concentration of VEGFR-3 at baseline had a better prognosis, indicating that these factors may be utilized as prognostic indicators for this sort of treatment (Rini et al., 2008).

RCC cells have high levels of the CIAX enzyme, which is activated by hypoxia. During hypoxic circumstances, this enzyme maintains tumor pH homeostasis by regulating the change of carbon dioxide to carbonic acid leading to lactic acid accumulation, which is correlated with cancer aggressiveness (Fig. 5) (Garcia et al., 2009, Lee and Griffiths, 2020).

There are other biomarkers also which can be used as indicative and predictive markers for kidney cancers such as HIF-1, Survivin, mTOR, CA9, PTEN, tyrosine kinases Akt, S6K, cytokines CCL5, CXCL9, and caveolin-1, but, due to their less specificity, they still have a limited usage (Mickley et al., 2015).

The HRAS gene was discovered in a BC cell line (Feinberg et al., 1983). According to recent studies, RAS gene mutations are found in just 1–13% of BC and are much less common in muscle-invasive cancers (Jebar et al., 2005, Sfakianos et al., 2015). Furthermore, the presence of a RAS gene mutation was not linked to disease-specific survival (Kompier et al., 2010). Therefore, their use as diagnostic or prognostic marker is not yet clear.

Mutations in fibroblast growth factor receptor 3 (FGFR3) gene are common in BC as non-muscle-invasive bladder cancer showed around 60–70% of mutation rate (Billerey et al., 2001, Cappellen et al., 1999). FGFR3 is essential for the progression of BC from the low stage (van Rhijn et al., 2012), described by low protein production and elevated cell cycle gene action (Lindgren et al., 2006, van Rhijn et al., 2012). However, at the time of diagnosis, its mutation is relatively less frequent in individuals with muscle-invasive BC and has not been proven as a predictive biomarker in advanced BC (Nagata et al., 2016).

TP53 is a transcription factor that controls apoptosis, cell proliferation, and cell cycle arrest (Nagata et al., 2016). TP53 nuclear accumulation is linked with a poorer prognosis in patients with advanced BC (Malats et al., 2005). TP53 expression was shown to be independently related to cancer recurrence and cancer-specific death in the variable analysis of 692 patients with aggressive tumors managed by cystectomy and lymphadenectomy (Shariat et al., 2010). Furthermore, 53% of patients who have had a radical cystectomy had the TP53 gene mutation, which wasrelated to a poor prognosis (Karam et al., 2007).

Other molecular biomarkers that are involved in the advanced level of BC progression are ERBB2/HER2 (Bolenz et al., 2010, Eissa et al., 2005), Loss of RB1 gene expression (Shariat et al., 2004),

and PTEN (Platt et al., 2009). This indicates that the signalling pathway from tyrosine kinase receptors to AKT/PI3K is likely to perform a significant role in the growth of BC and other malignancies. Although, many biomarkers have been identified for BC but for their clinical implication, more in-depth analysis would be required (Nagata et al., 2016).

### 6.3 Liquid molecular biomarkers

The liquid molecular biomarkers of interest in genitourinary oncology include the circulating tumor cells (CTCs), the circulating tumor DNA (ctDNA), cell-free DNA (cfDNA), RNAs, proteins, metabolites, and extracellular vesicles (Lodewijk et al., 2018). At the same time, serum and plasma are most often utilized for liquid biopsies to identify and monitor BC. Furthermore, a liquid biopsy sample widely used for detection and surveillance is urine because of its close proximity to the tumor. Moreover, exfoliated cells produced from cancer can be detected in the urine (Lodewijk et al., 2018).

CTCs are one of the most significant classes of the rare circulating cells which are subjected to liquid biopsy. The procedure has obtained validation and approval by the Food and Drug Administration (FDA) as a helpful strategy to track the prognosis of various types of cancers (Harouaka et al., 2014). This strategy is based on the ability of the CTCs in mirroring the tumor heterogeneity and the potential to use the single CTCs in an epigenomic analysis by combining their genetic & transcriptional statuses (Harouaka et al., 2014). In recent times, CTCs have gained good recognition due to their possible roles in evaluating the status of limited-spread and metastatic cancers. However, this cell-based diagnostic is not in frequent use globally, despite the requisite FDA approvals. The liquid molecular biomarker CTCs have applications in all types of GU cancers (Lodewijk et al., 2018).

Cell search technology is the sole method for detecting CTCs in BC and PC patients. This technology helped in detection of CTCs in as many as 57% of the total subjects suffering from PC (Allard et al., 2004). Baseline value of CTCs below five cells for each 7.5 ml of blood was found to have a statistically significant association with a good OSR of 21.70 months compared to 11.50 months in those having less than five cells in a similar volume of blood (de Bono et al., 2008). Furthermore, a reduction in CTCs while therapy is underway and immediately after has been linked with an improved survival rate (Yan et al., 2017).

In the case of RCC, non-invasive biomarkers like CTCs can be used to customize therapeutic changes at an earlier stage (Klezl et al., 2020). Strategies are underway to use these non-invasive biomarkers for developing detection/analysis methods and introduce these analytical techniques for CTCs in routine care of patients with RCC. It is foreseen that in the years to come, the entire therapeutic process for RCC will be revolutionized with the incorporation of the prognostic use of CTCs in therapeutics for RCC (Klezl et al., 2020). Some methods which collect, identify, enrich, and analyze the CTCs are using the Epithelial Cell Adhesion Molecules (EpCam) epithelial biomarker, which belong to the family of transmembrane glycoproteins having the function in various cellular signalling pathways (Maetzel et al., 2009). It has been shown that EpCam is also involved in cellular motility, including migration, proliferation, and differentiation (Maetzel et al., 2009).

The other liquid molecular biomarker that can be used in prostate cancer is ctDNA. Genomic mutation of the ctDNA can be evaluated by repeatedly analyzing ctDNA, leading to a great extent of concordance with the assessed primary lesions and metastasized lesions (Cheng et al., 2016). The phenomenon of detecting mutation in the DNA repair genes in the ctDNA is gaining an escalating interest of medical scientists due to the potential benefits of the therapy. For example, targeting DNA damage repair enzyme “poly adenosine diphosphate-ribose polymerase”(PARP) (Heale et al., 2006) for improving reproductive system cancers treatment has been undertaken as therapeutics (O’Sullivan Coyne et al., 2017).

Small non-coding RNAs (sncRNAs) in urine have been utilized as non-invasive biomarkers for BC, but their application in plasma has yet to be thoroughly investigated (Guo et al., 2021). Next-generation sequencing in BC was used to examine the expression levels of sncRNAs in plasma extracellular vesicles. It was found that sncRNAs such as miR-4508, miR-126-3p, miR-185-5p, miR-106a-5p, and miR-10b-5p from plasma extracellular vesicles can be used as diagnostic biomarkers (Sabo et al., 2020).

It has been found that aggressive malignant tumors harbour Moesin (MSN) molecule. MSN gene was found to be overexpressed in BC and was correlated with cellular invasion (Park et al., 2020). Interestingly, there was a significant decrease in cell invasion after inhibiting MSN in BC, suggesting that MSN gene might serve as a diagnostic biomarker for BC invasiveness (Park et al., 2020).

The measurement of DNA methylation in urine samples has also been used to for detection and surveillance of BC. The DNA methylation was studied in three samples of urine viz. full void urine, urine pellet and supernatant to evaluate which urine fraction could be used for BC diagnosis based on methylation markers. According to the findings of the study, both cellular and cell-free DNA in urine can be utilized for methylation analysis in BC, with urine pellets serving as the most appropriate fraction for the analysis (Hentschel et al., 2020).

The most efficient testing for BC is through the urine. Although several urinary tests are available and have the requisite FDA approvals, how accurate, specific, and sensitive these tests are, is still questionable. It has resulted in the need for the development of biomarker-based diagnostic methods for BC. For this reason, much scientific work is underway to tap the advantage of DNA-based & RNA-based biomarkers in routine oncology practice for BC (Lopez-Beltran et al., 2020).

## 7 Targeting biomarkers for genitourinary cancers treatment

Similar to the evolution of diagnostic techniques used for the diagnosis and tracking the prognosis of genitourinary cancers, the treatment of these cancers has evolved significantly in the last few years. Although the cancers included in the broad classification of GU cancers like bladder, prostate, and kidney cancers vary considerably in their etiology, pathophysiology, and the required diagnostic workup, all of these have undergone significant transitions as far as their treatment modalities are concerned. The underlying pathophysiological mechanisms have been better understood over the years. Many new molecular-level mechanisms have been discovered that have led to the evolution of advanced diagnostic and therapeutic strategies (Zarrabi et al., 2019).

Immunotherapy has emerged as one of the most studied and successful therapeutic strategy against GU cancers. In the broader domain of immunotherapy, immune checkpoint inhibitors have revolutionized the treatment approach for GU cancers. Novel therapeutic agents have been developed within this broad domain of immunotherapeutics to cure these cancers. Although the precise mechanism of action within this class of medications differs between the individual therapeutic agents, they all have a similar broader mechanism of action which involves modulating the immune response within the pathophysiology of the tumor (Zarrabi et al., 2019).

Conventionally, immune therapy has long been used for GU cancers. Immunotherapy with interleukin-2 and interferon-γ has been implicated in case of urothelial and kidney cancers. It acts by stimulating T-lymphocytes prior to attacking cancer cells (Huang and Hsieh, 2020). However, the therapeutic response to these immunological agents has not been fully understood (Donskov et al., 2018, Schmitz et al., 2020). The failure of conventional immunological agents instigated the research for novel agents (Zarrabi et al., 2019). The quest for newer, more effective treatment modalities has proven to be successful, as evidenced by the development of novel immune checkpoint inhibitors that have transformed the treatment of GU cancers (Balar et al., 2017, Bellmunt et al., 2017).

The emerging therapies for BC include immune checkpoint inhibition, mono- and combination therapies. These mono- and combination therapies include cytokines modulation, inhibition of transforming growth factor-beta (TGFβ), 4-1BB, OX-40, and IDO. Furthermore, VEGFR, nectin, human growth factor, and FGFR inhibitors have also been used as therapeutic agents (Zarrabi et al., 2019). For RCC, the emerging therapies include the use of novel anti-angiogenesis therapies. For instance, vascular endothelial and tyrosine kinase pathways are targeted by cabozantinib inhibitor or by combining nivolumab (anti-PD-1) and ipilimumab (anti-CTLA-4) inhibitors (Hudes et al., 2007, Motzer et al., 2007, Sternberg et al., 2010). The focus of novel agents in the case of PC is on metastatic castration-resistant prostate carcinoma (mCRP). For this type of cancer, the emerging therapeutic agents include PARP inhibitors which have served as promising treatment strategy (Pezaro, 2020).

The inhibitors of PD-1 and PD-L1 have received FDA approval to treat metastatic urothelial and RCC and have demonstrated increased survival rate in these patients (Balar et al., 2017, Rosenberg et al., 2016). Researchers have identified that the microbiome in the gut is closely related to the overall body metabolism and the immunological system in the pathogenesis of cancer. A specific example is that of RCC, in which the conformation of the gut microbiome can impact the resistance to blockage of the PD-1 pathway (Xu et al., 2020). Therefore, antibiotic treatment inhibits the beneficial effect of immunotherapeutic agents in subjects with advanced-stage cancers and is representative of the resistance to the blockage of the PD-1 pathway (Xu et al., 2020).

Research on the microbiomes of subjects having BC and PC has demonstrated substantial differences in the composition of bacteria compared to the controls that do not have the condition. Furthermore, in the case of PC, the detection of metabolic products derived from the microbiome is substantially different from the controls. These differences can be used to develop microbiome-based profiles to predict the risk groups for PC (Cavarretta et al., 2017).

It could be concluded that the therapeutic techniques hold a great promise for application in treatment, prediction of disease, therapeutic outcomes, and prognosis in many GU cancers, including those of the kidneys, urinary bladder, and prostate glands (Montironi et al., 2016).

## 8 Conclusion

Based on the information discussed in this review, it can be concluded that GU cancers hold a unique position as a group of cancers due to their high prevalence in male subjects. The diagnostic and therapeutic processes for GU cancers are different from other cancers. As reviewed, much research has been conducted on GU cancers in recent years which has provided a massive base of knowledge about the promising diagnostic and therapeutic approaches for these cancers. The most significant scientific development in genitourinary oncology is the application of biomarkers that can be used to diagnose, track, and treat various types of GU cancers. Several biomarkers are available to the physicians in the contemporary world to help detection of these cancers at an early phase and with more accuracy. Many new biomarkers are being evolved, and it seems that disease outcomes for these tumors will be improved dramatically in the following years. Novel therapies based on immunological techniques have already emerged and are revolutionizing the science of uro-oncology.

The field of genitourinary oncology has a promising future, and continuing biomarker validation and optimization in conjunction with combination therapy may help to increase the effectiveness of the treatment. Overall, biomarkers hold an important role in the diagnosis, treatment, and prognosis of GU malignancies.

## Declaration of Competing Interest

The authors declare that they have no known competing financial interests or personal relationships that could have appeared to influence the work reported in this paper.
